# IS*26* Is Responsible for the Evolution and Transmission of *bla*_NDM_-Harboring Plasmids in Escherichia coli of Poultry Origin in China

**DOI:** 10.1128/mSystems.00646-21

**Published:** 2021-07-13

**Authors:** Qiu-Yun Zhao, Jia-Hang Zhu, Run-Mao Cai, Xing-Run Zheng, Li-Juan Zhang, Man-Xia Chang, Yue-Wei Lu, Liang-Xing Fang, Jian Sun, Hong-Xia Jiang

**Affiliations:** a Guangdong Provincial Key Laboratory of Veterinary Pharmaceutics Development and Safety Evaluation, College of Veterinary Medicine, South China Agricultural University, Guangzhou, China; b Guangdong Laboratory for Lingnan Modern Agriculture, Guangzhou, China; Agricultural Biotechnology Research Center

**Keywords:** *bla*_NDM_, IS*26*, circular intermediate, recombination, evolution, plasmid, *Escherichia coli*

## Abstract

Carbapenem-resistant Enterobacteriaceae are some of the most important pathogens responsible for nosocomial infections, which can be challenging to treat. The *bla*_NDM_ carbapenemase genes, which are expressed by New Delhi metallo-β-lactamase (NDM)-producing Escherichia coli isolates, have been found in humans, environmental samples, and multiple other sources worldwide. Importantly, these genes have also been found in farm animals, which are considered an NDM reservoir and an important source of human infections. However, the dynamic evolution of *bla*_NDM_ genetic contexts and *bla*_NDM_-harboring plasmids has not been directly observed, making it difficult to assess the extent of horizontal dissemination of the *bla*_NDM_ gene. In this study, we detected NDM-1 (*n* = 1), NDM-5 (*n* = 24), and NDM-9 (*n* = 8) variants expressed by E. coli strains isolated from poultry in China from 2016 to 2017. By analyzing the immediate genetic environment of the *bla*_NDM_ genes, we found that IS*26* was associated with multiple types of *bla*_NDM_ multidrug resistance regions, and we identified various IS*26*-derived circular intermediates. Importantly, in E. coli strain GD33, we propose that IncHI2 and IncI1 plasmids can fuse when IS*26* is present. Our analysis of the IS*26* elements flanking *bla*_NDM_ allowed us to propose an important role for IS*26* elements in the evolution of multidrug-resistant regions (MRRs) and in the dissemination of *bla*_NDM_. To the best of our knowledge, this is the first description of the dynamic evolution of *bla*_NDM_ genetic contexts and *bla*_NDM_-harboring plasmids. These findings could help proactively limit the transmission of these NDM-producing isolates from food animals to humans.

**IMPORTANCE** Carbapenem resistance in members of the order Enterobacterales is a growing public health problem that is associated with high mortality in developing and industrialized countries. Moreover, in the field of veterinary medicine, the occurrence of New Delhi metallo-β-lactamase-producing Escherichia coli isolates in animals, especially food-producing animals, has become a growing concern in recent years. The wide dissemination of *bla*_NDM_ is closely related to mobile genetic elements (MGEs) and plasmids. Although previous analyses have explored the association of many different MGEs with mobilization of *bla*_NDM_, little is known about the evolution of various genetic contexts of *bla*_NDM_ in E. coli. Here, we report the important role of IS*26* in forming multiple types of *bla*_NDM_ multidrug resistance cassettes and the dynamic recombination of plasmids bearing *bla*_NDM_. These results suggest that significant attention should be paid to monitoring the transmission and further evolution of *bla*_NDM_-harboring plasmids among E. coli strains of food animal origin.

## INTRODUCTION

Infection with carbapenemase-producing Enterobacteriaceae (CPE) strains is associated with high mortality rates because therapeutic options are limited. New Delhi metallo-β-lactamases (NDMs), a class of carbapenemases that hydrolyze virtually all β-lactams, are one of the most important resistance traits in Escherichia coli (1). Since *bla*_NDM-1_ was initially identified in Klebsiella pneumoniae in 2009, *bla*_NDM_ genes have been reported to be abundant worldwide, and the frequency of these genes in clinical isolates in Asia is increasing at an alarming rate (2–4). Although the rapid spread of NDM-encoding genes has gained global attention (5, 6), to date no study has focused on the dynamic evolution of the *bla*_NDM_ genetic context.

The dissemination of NDM-1 mainly involves plasmids rather than clonal spread. In E. coli isolates, *bla*_NDM_ has been identified on plasmids with a narrow (IncFIB and IncFII) or broad (IncX3, IncA/C, IncH, IncL/M, and IncN) host range (7, 8). Moreover, mobile genetic elements (MGEs) can facilitate the spread of *bla*_NDM_ genes, and the similarity of the *bla*_NDM_-flanking sequences in these plasmids suggests that horizontal mobilization of *bla*_NDM_ via MGEs is responsible for its evolution and rapid transmission between plasmids and chromosomes (8–10). Furthermore, insertion sequences, such as IS*Aba125*, IS*3000*, IS*26*, IS*5*, IS*CR1*, Tn*3*, Tn*125*, Tn*3000*, and Tn*1548*, seem to play an important role in the dissemination of NDM-encoding genes (8, 11–13). Most of the *bla*_NDM_ sequences available in GenBank are flanked upstream by a complete or truncated copy of IS*Aba125* and downstream by the *ble*_MBL_ gene. Many different MGEs have been found bracketing these genes that could potentially mobilize them (14).

Despite the fact that carbapenems are rarely used in animals (9, 10, 15), NDM-producing E. coli strains have been isolated from food animals in Asia, suggesting that food animals are another important reservoir of NDM-producing strains (16–18). Farmed poultry are a particularly common source of NDM-positive E. coli, raising the possibility of food chain transmission, which further highlights the importance of controlling the spread of NDMs. Therefore, to assess the mechanism by which the *bla*_NDM_ gene is transferred among E. coli strains isolated from poultry, the genomes of *bla*_NDM_-positive E. coli isolates from chickens and ducks were analyzed to determine how MGEs contribute to the generation of *bla*_NDM_-harboring plasmids. We found various IS*26*-flanked pseudo-compound transposons (PCTs) (19) and, furthermore, fusion and resolution plasmids that had formed when IS*26* is present in both IncHI2 and IncI1 plasmids. Our analysis allowed us to propose the contribution to evolution of MRRs and dissemination of *bla*_NDM_ when mobile element IS*26* is present.

## RESULTS

### NDM-producing E. coli isolates.

**FIG 1:**
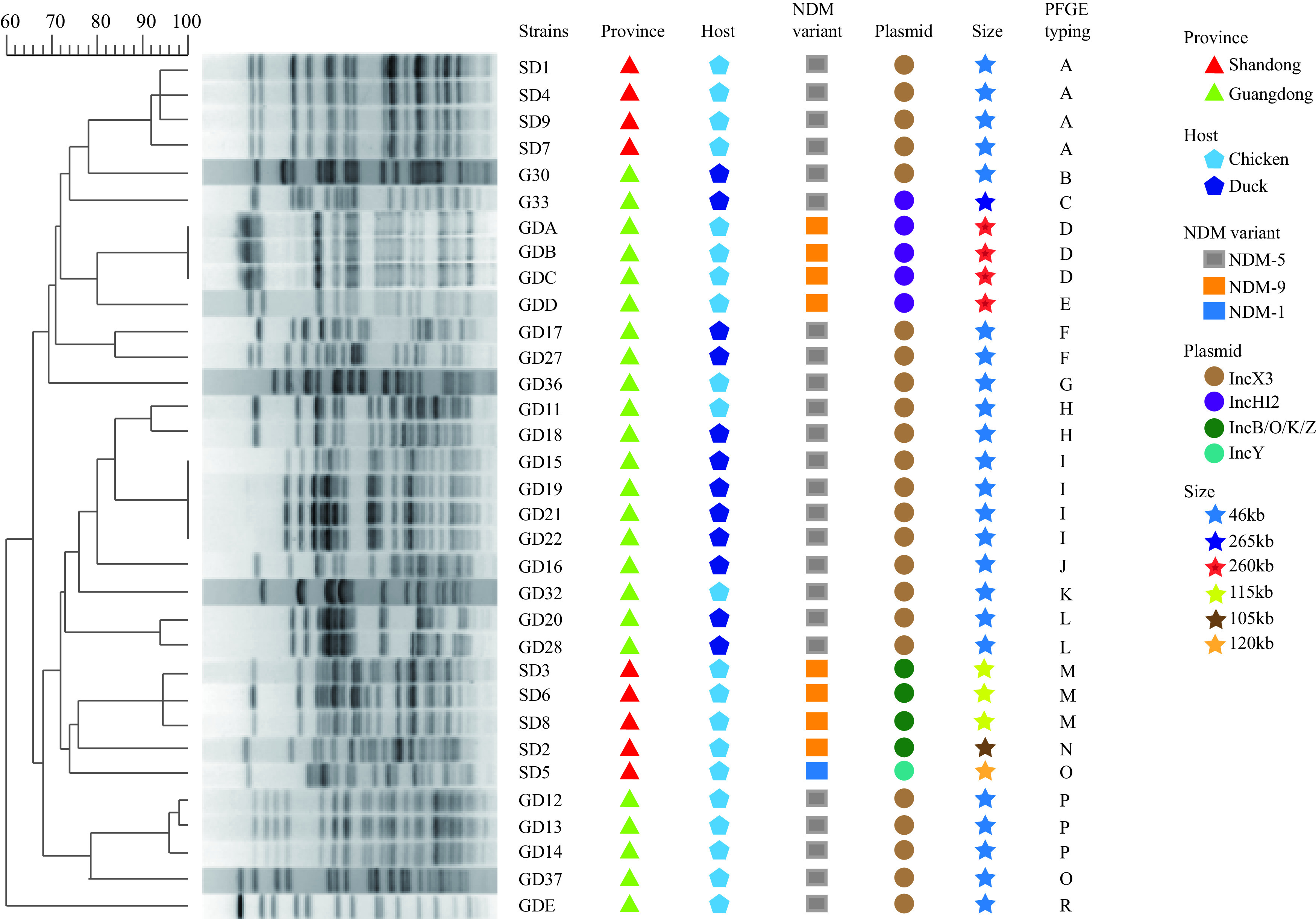
Pulsed-field gel electrophoresis (PFGE) patterns and resistance genes of 33 *bla*_NDM_-harboring Escherichia coli strains.

A total of 470 E. coli isolates were obtained from 470 samples collected from six poultry farms in Guangdong Province (four chicken farms and one duck farm) and Shandong Province (one chicken farm). In total, 33 (7.02%) of these E. coli isolates carried *bla*_NDM_ genes (Fig. 1; see also Table S1 in the supplemental material). The prevalence of *bla*_NDM_-positive E. coli isolates in Shandong and Guangdong Provinces was 25.71% (9/35) and 5.52% (24/435), respectively. The *bla*_NDM_-positive E. coli strains were isolated from both chickens (5.34%, 21/393) and ducks (15.58%, 12/77). NDM-5 (72.7%, 24/33) was the predominant variant, followed by NDM-9 (24.2%, 8/33) and NDM-1 (3.0%, 1/33) (Fig. 1). All 33 of the *bla*_NDM_-positive E. coli isolates showed multidrug resistance (MDR), as determined by testing against a panel of 20 antimicrobial agents (Table S1). The 33 *bla*_NDM_-positive E. coli strains were grouped into 19 clusters based on XbaI pulsed-field gel electrophoresis (PFGE) sequence similarity (with a threshold of 85% similarity), suggesting that most of the strains were epidemiologically unrelated (Fig. 1). Each cluster carried the same NDM variant, while cluster H contained isolates GD11 and GD18, which appeared to be clonally related strains that were from different hosts (chicken and duck). Taken together, both horizontal transmission and clonal dissemination were responsible for the distribution of the *bla*_NDM_ gene.

10.1128/mSystems.00646-21.5 TABLE S1 MIC of *bla*_NDM_-harboring E. coli to different antibiotics. Download Table S1, DOCX file, 0.04 MB. Copyright © 2021 Zhao et al. 2021 Zhao et al. https://creativecommons.org/licenses/by/4.0/ This content is distributed under the terms of the Creative Commons Attribution 4.0 International license.

### Analysis of the localization of *bla*_NDM_.

To determine the location of *bla*_NDM_ genes, we performed conjugation/transformation, PCR-based replicon typing (PBRT), S1-PFGE, and Southern hybridization. There were four types of replicons carrying the *bla*_NDM_ gene, namely IncX3, IncB/O/K/Z, IncHI2, and IncY. *bla*_NDM-5_ was located on the IncX3 (23/33; 46-kb) and IncHI2 (1/33; 256-kb) plasmids, *bla*_NDM-9_ was located on the IncHI2 (4/33; 260-kb) and IncB/O/K/Z (4/33; 105to 115-kb) plasmids, and *bla*_NDM-1_ was located on the IncY (1/33; 120-kb) plasmid (Fig. 1).

### Characteristics of *bla*_NDM-9_-carrying IncB/O/K/Z plasmids.

Out of the four *bla*_NDM-9_-harboring IncB/O/K/Z plasmids that we isolated from E. coli strains, we sequenced two, pNDM-T2 (105-kb) and pNDM-T6 (115-kb). The complete sequence of pNDM-T2 is a 106,300-bp circular molecule with a GC content of 54% and was predicted to harbor 215 open reading frames (ORFs). The plasmid of pNDM-T6 was 115,219 bp with a GC content of 55% and was predicted to harbor 240 ORFs. The plasmid backbones of both pNDM-T2 and pNDM-T6 were highly similar (99% coverage and 100% identity) to that of the *bla*_NDM-9_-harboring IncB/O/K/Z plasmid pHNTH02-1 (GenBank accession number MG196294) isolated from chicken meat in China (see Fig. S1 in the supplemental material).

10.1128/mSystems.00646-21.1 FIG S1 Sequence alignment of *bla*_NDM-9_-positive IncB/O/K/Z plasmids. Download FIG S1, DOCX file, 0.1 MB. Copyright © 2021 Zhao et al. 2021 Zhao et al. https://creativecommons.org/licenses/by/4.0/ This content is distributed under the terms of the Creative Commons Attribution 4.0 International license.

**FIG 2:**
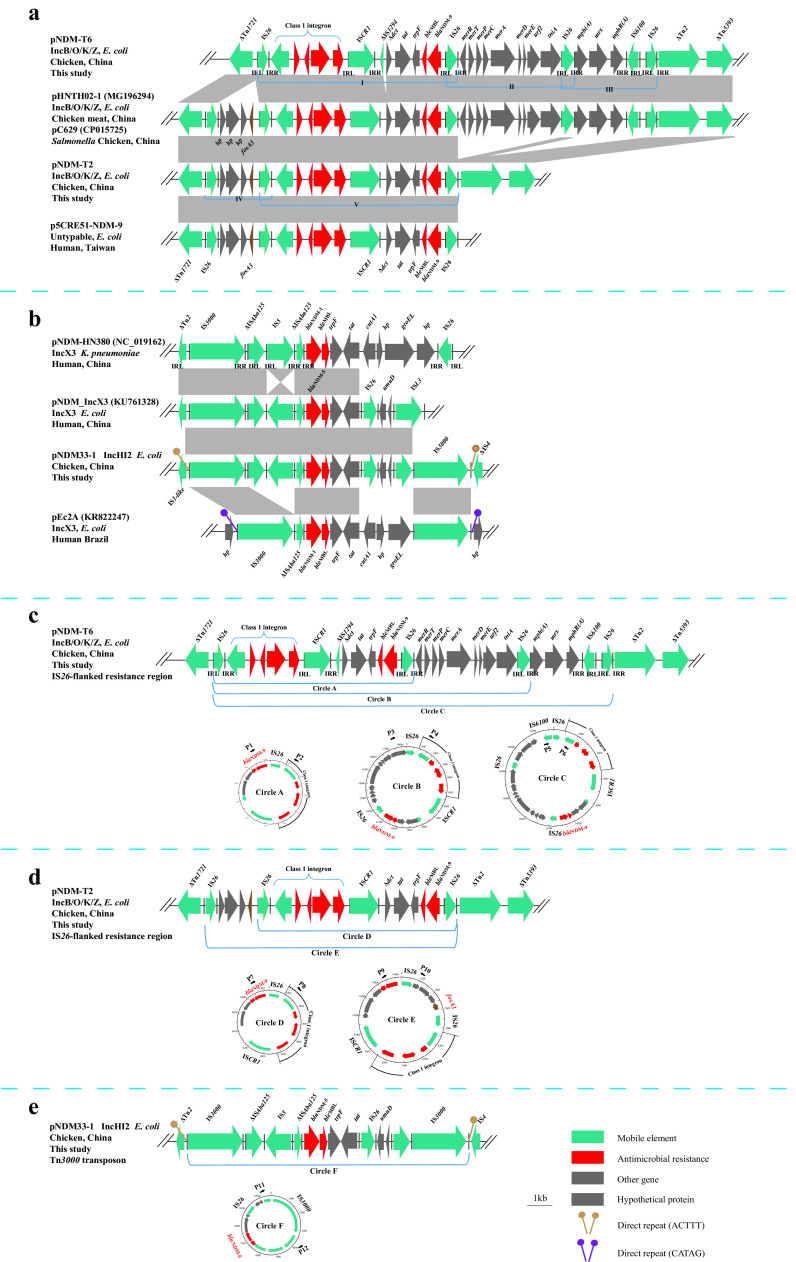
Genomic and molecular analyses of the *bla*_NDM-9_-positive IncB/O/K/Z plasmids and the *bla*_NDM-5_-positive IncHI2 plasmids. (a) Genetic features of *bla*_NDM-9_ on IncB/O/K/Z. (b) Genetic features of *bla*_NDM-5_ on IncHI2. (c) Schematic representation of the circular forms obtained from pNDM-T6, as assessed by PCR and sequencing. (d) Schematic representation of the circular forms obtained from pNDM-T2, as assessed by PCR and sequencing. (e) Schematic representation of the circular forms obtained from pNDM33-1, as assessed by PCR and sequencing. The arrows indicate the positions and directions of transcription for the genes. Regions of >99.0% nucleotide sequence identity are shaded in gray. The delta (Δ) symbol indicates a truncated gene. IRL, terminal inverted repeat, left; IRR, terminal inverted repeat, right; *hp*, hypothetical protein.

In pNDM-T6, the MRR bounded by Tn*1721* and ΔTn*5393* was interspersed with a number of different resistance genes, including *fosA3*, *dfrA12*, *aadA2*, *sul1*, *ble*_MBL_, *mph*(A), and *mer*, as well as several mobile elements, including IS*26*, IS*CR1*, ΔIS*Aba125*, ΔIS*5075*, IS*6100*, and ΔTn*2*. The *bla*_NDM-9_ gene was embedded in a 27.5-kb IS*CR1* complex class 1 integron (Fig. 2a). This region was bracketed by two IS*26* elements in the same orientation. Similar structures have also been reported in E. coli plasmid pHNTH02-1 (GenBank accession number MG196294) and Salmonella plasmid pC629 (GenBank accession number CP015725) (20).

The pNDM-T6 hypervariable region in this MRR consisted of four IS*26* elements flanking the following three different segments: (i) IS*26*-*IntI1*-*dfrA12*-*aadA2*-*qacE*Δ*1*-*sul1*-IS*CR1*-ΔIS*Aba125*-Δ*dct*-*tat*-*trpF*-*ble*_MBL_-*bla*_NDM-9_-IS*26*, (ii) IS*26*-*merR*-*merT*-*merP*-*merC*-*merA*-*merD*-*merE*-*urf2*-*tniA*-IS*26*, and (iii) IS*26*-*mph*(A)-*mrx*-*mphR*(A)-IS*6100*-IS*26* (Fig. 2c). In pNDM-T2, the MRR consisted of three IS*26* elements flanking two different segments, as follows: (iv) IS*26*-*hp*-*hp*-*hp*-*fosA3*-IS*26* and (v) IS*26*-*IntI1*-*dfrA12*-*aadA2*-*qacE*Δ*1*-*sul1*-IS*CR1*-Δ*dct*-*tat*-*trpF*-*ble*_MBL_-*bla*_NDM-9_-IS*26* (Fig. 2d). This MRR shared 100% identity with the corresponding segment in the p5CRE51-NDM9 (GenBank accession number CP021177) plasmid from an E. coli strain isolated from human urine (21).

To test and verify circular intermediates formed, reverse PCR using the primers shown in Table S2 in the supplemental material was performed with pNDM-T2 and pNDM-T6 as the template DNA. Five circular intermediates of the four IS*26*-flanked PCTs were identified (Fig. 2c and d). Sequence analysis showed that each circular intermediate contained only one copy of IS*26*, which suggests how IS*26* facilitates construction of circular intermediates. These findings indicated that IS*26*-flanked PCTs are dynamic and that the circular intermediate could readily be excised from the plasmid, thereby facilitating its transposition into other plasmids, confirming how IS*26* mediates transposition of antibiotic resistance genes (ARGs).

10.1128/mSystems.00646-21.6 TABLE S2 Primers used to detect *bla*_NDM_-harboring transconjugants. Download Table S2, DOCX file, 0.02 MB. Copyright © 2021 Zhao et al. 2021 Zhao et al. https://creativecommons.org/licenses/by/4.0/ This content is distributed under the terms of the Creative Commons Attribution 4.0 International license.

### Characteristics of IncX3 plasmids carrying *bla*_NDM-5_.

Out of the 23 *bla*_NDM-5_-harboring IncX3 plasmids (46 kb) isolated from E. coli strains, we randomly selected pNDM-T16 from GD16 for Illumina sequencing. The complete sequence of pNDM-T16 is a 46,161-bp circular molecule with a GC content of 47% and was predicted to harbor 100 open reading frames (ORFs). BLAST homology analysis showed that pNDM-T16 was highly homologous (100% coverage and 99.99% identity) to the *bla*_NDM-5_-harboring IncX3 plasmid pNDM5_IncX3 (GenBank accession number KU761328) from a Klebsiella pneumoniae clinical isolate from China (22). Taken together, these findings suggest that similar IncX3 plasmids were most likely responsible for spreading *bla*_NDM-5_ in Enterobacteriaceae.

### Fusion of plasmid pNDM33-1 and pNDM33-2.

**FIG 3:**
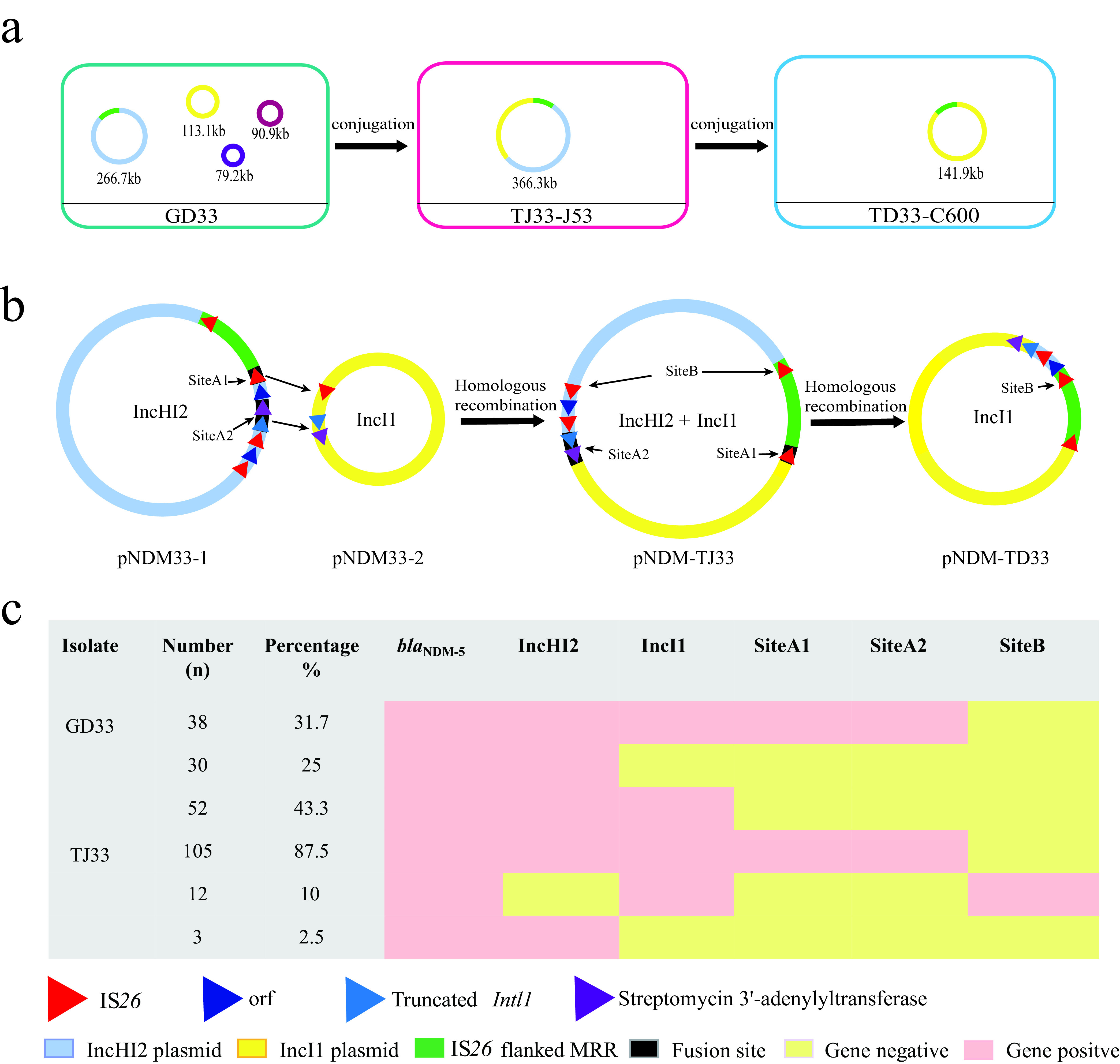
Mechanisms of plasmid fusion. (a) Sizes of the plasmids isolated from each strain. (b) Homologous recombination. (c) Detection of recombination types in strains GD33 and TJ33.

To determine why *bla*_NDM-5_ was located on the 265-kb IncHI2 plasmid in parental strain GD33 but was located on a 366-kb plasmid in the transconjugant TJ33 when conjugated with the recipient strain E. coli J53, we performed whole-genome sequencing of GD33 and TJ33. Sequence analysis revealed that there were four plasmids of different sizes (266,777 bp, 113,068 bp, 90,896 bp, and 79,203 bp) in GD33, but only one plasmid (366,267 bp) in the transconjugant TJ33 (Fig. 3a). The pNDM33-1 plasmid was 266,777 bp with a GC content of 47.07%, predicted to harbor 339 ORFs, and belonged to the IncHI2 incompatibility type. BLAST analysis showed that the plasmid had high homology with pTB-nb4 (GenBank accession number CP033636; 99% coverage and 99.98% identity), which was isolated from an E. coli strain isolated from chicken in China. On pNDM33-1, the 13,918-bp Tn*3000* transposon unit (IS*3000*-ΔIS*Aba125*-IS*5*-ΔIS*Aba125*-*bla*_NDM-5_-*ble*_MBL_-*trpF-tat*-*dct*-IS*26*-*umuD*-IS*L3*-IS*3000*) inserted between IS*1* family transposon and IS*4*-like element genes was flanked by 5-bp direct repeats (DRs) (ACTTT), suggesting insertion of the Tn*3000* transposon unit (see Fig. S2 in the supplemental material). This transposon unit was highly similar to that reported in the IncX3 plasmid pNDM5_IncX3 (GenBank accession number KU761328) isolated from a clinical E. coli strain (23), except for absence of IS*3000* downstream of *bla*_NDM-5_ (Fig. 2b). A similar Tn*3000* transposon unit harboring *bla*_NDM-1_ in an IncX3 plasmid pEc2A (GenBank accession number KR822247) was recently reported in a clinical E. coli strain from Brazil (13). This transposon unit was also identified in an IncX3 plasmid, pNDM-HN380 (GenBank accession number NC_019162) from Klebsiella pneumoniae of human origin in China. One circular form (F) was detected by reverse PCR and Sanger sequencing (see Table S2 in the supplemental material). This circular form contained only one copy of IS*3000*, which suggests where the circular form excised from the plasmid of pNDM33-1 (Fig. 2E). pNDM33-2 was 113,068 bp in size, had a GC content of 50%, was predicted to harbor 157 ORFs, belonged to the IncI1 incompatibility group, and carried *erm*(B), *mph*(A), *floR*, and *aadA22* resistance genes. BLAST analysis showed that this type of plasmid has previously been reported in E. coli and Salmonella; pS68 (GenBank accession number KU130396; 93% coverage and 99.78% identity) was isolated from an E. coli strain in China, and pUY_STM96 (GenBank accession number MN241905; 86% coverage and 99.9% identity) was isolated from a clinical Salmonella enterica subsp. *enterica* serovar Typhimurium strain in Uruguay.

10.1128/mSystems.00646-21.2 FIG S2 Sequence alignment of *bla*_NDM-5_-positive IncHI2 plasmids. Download FIG S2, DOCX file, 0.1 MB. Copyright © 2021 Zhao et al. 2021 Zhao et al. https://creativecommons.org/licenses/by/4.0/ This content is distributed under the terms of the Creative Commons Attribution 4.0 International license.

The largest plasmid, pNDM-TJ33, that was obtained from transconjugant TJ33 was 366,267 bp in size with 485 ORFs, belonged to the IncI1 and IncHI2 plasmid families, and had a GC content of 47.67% (see Fig. S3 in the supplemental material). Detailed sequence analysis of these three plasmids (pNDM33-1, pNDM33-2, and pNDM-TJ33) enabled us to predict the possible mechanism of plasmid fusion. The IncHI2 and IncI1 plasmids both contain the same IS*26*, streptomycin 3′-adenylyltransferase, and truncated *IntI1* gene sequences (Fig. 3b). We named the fusion sites between pNDM33-1 and pNDM33-2 site A1 and site A2. The fusion plasmid, pNDM-TJ33, exhibits a significantly broadened resistance profile, covering several additional classes of antibiotics, including aminoglycosides, beta-lactams, quinolones, tetracyclines, and macrolides (see Table S3 in the supplemental material).

10.1128/mSystems.00646-21.3 FIG S3 Pairwise BLASTn alignment of pNDM33-1, pNDM33-2, pNDM-TJ33, and pNDM-TD33, performed using BRIG. Download FIG S3, DOCX file, 0.3 MB. Copyright © 2021 Zhao et al. 2021 Zhao et al. https://creativecommons.org/licenses/by/4.0/ This content is distributed under the terms of the Creative Commons Attribution 4.0 International license.

10.1128/mSystems.00646-21.7 TABLE S3 Distribution of resistance genes and replicons in four plasmids related to plasmid homologous recombination. Download Table S3, DOCX file, 0.02 MB. Copyright © 2021 Zhao et al. 2021 Zhao et al. https://creativecommons.org/licenses/by/4.0/ This content is distributed under the terms of the Creative Commons Attribution 4.0 International license.

### Resolution of plasmid pNDM-TJ33.

To test whether the fusion plasmid could be mobilized, a conjugation assay was performed with TJ33 as the donor strain and streptomycin-resistant E. coli C600 as the recipient strain. This yielded a *bla*_NDM-5_-carrying plasmid, pNDM-TD33 (141,890 bp), which was IncI1 type, had a GC content of 50.32%, and was predicted to harbor 193 ORFs. Alignment of pNDM-TD33 with pNDM-TJ33 showed that pNDM-TD33 was derived from pNDM-TJ33, and pNDM-TD33 was excised at this homologous sequence, IS*26* (named site B), as shown in Fig. 3b. The new plasmid, pNDM-TD33, belonged to the IncI1 replicon group and contained most of pNDM33-2, as well as two IS*26* elements flanking the *bla*_NDM-5_-harboring fragment from pNDM33-1 (Fig. 3b). Significantly, pNDM-TD33 seems to have obtained the carbapenem resistance gene *bla*_DNM-5_ from pNDM33-1 (Table S3).

### Dynamic balance of plasmid fusion and resolution.

A recently reported example shows that IS*26* actively remodels resistance plasmids via both inter- and intramolecular replicative transposition (24). To determine whether the same mechanism was active among pNDM33-1, pNDM33-2, and pNDM-TJ33, we performed a conjugation assay using GD33 and TJ33 as the donor strains and azide-resistant E. coli J53 and streptomycin-resistant E. coli C600 as the recipient strains. A total of 120 carbapenem-resistant transconjugants from each conjugation were randomly selected and screened for the presence of sites A1, A2, and B using the primers shown in Table S2 (Fig. 3b). We also assessed the presence or absence of *bla*_NDM-5_, as well that of as the IncHI2 and IncI1 replicons. Interestingly, 38 (31.7%) of the GD33 transconjugants (TJ33) contained all of the targets except for site B, 30 (25%) only carried *bla*_NDM-5_ and the IncHI2 replicon, and 52 (43.3%) were negative for site A1, A2, or both (Fig. 3c). Twelve (10%) of the 120 TJ33 transconjugants (TD33) were positive for site B, *bla*_NDM-5_, and the IncI1 replicon, and 105 (87.5%) transconjugants were positive for sites A1 and A2, *bla*_NDM-5_, and the IncHI2 and IncI1 replicons. These results suggest that new plasmid formation could occur in the parent strains and that there was a dynamic balance of recombination in the host bacteria.

### Plasmid stability and fitness.

To explore the transmissibility of *bla*_NDM-5_-carrying plasmids, the conjugation frequency of *bla*_NDM-5_-carrying plasmids was assessed. The conjugation frequency of pNDM33-1 (recipient strain E. coli J53) and pNDM-TJ33 (recipient strain E. coli C600) at 37°C were 1.00 × 10^−3^ to 2.61 × 10^−3^ and 1.79 × 10^−3^ to 4.09 × 10^−3^, respectively, while the conjugation frequency of pNDM-TD33 (recipient strain E. coli J53) was 1.09 × 10^−2^ to 1.74 × 10^−2^. Additionally, stability experiments performed in the absence of antibiotics demonstrated that pNDM-TD33 was much more stable than pNDM33-1 and pNDM-TJ33 (see Fig. S4a in the supplemental material). Strains carrying pNDM33-1, pNDM-TJ33, or pNDM-TD33 had fitness values that ranged between 0.81 and 1.44 in the absence of antibiotic pressure. pNDM-TD33 had a fitness value greater than 1, representing a significant increase in E. coli fitness (Fig. S4b).

10.1128/mSystems.00646-21.4 FIG S4 Effects of pNDM33-1, pNDM-TJ33, and pNDM-TD33 on E. coli host strains. (a) Plasmid stability. (b) Competition. Download FIG S4, DOCX file, 0.1 MB. Copyright © 2021 Zhao et al. 2021 Zhao et al. https://creativecommons.org/licenses/by/4.0/ This content is distributed under the terms of the Creative Commons Attribution 4.0 International license.

## DISCUSSION

In this study, we investigated the dynamic evolution of the *bla*_NDM_ genetic context in E. coli isolates from farmed poultry in China. Our results revealed that the genetic context of *bla*_NDM_ contains multiple IS*26*-flanked PCTs and that a dynamic balance of fusion and resolution of plasmids containing *bla*_NDM_ occurs when IS*26* is present. Taken together, our findings suggest that IS*26* contributes to formation of *bla*_NDM_ multidrug resistance cassettes and actively remodels antibiotic resistance plasmids.

IS*26*, which is frequently associated with genes encoding antibiotic resistance factors, has been reported to flank PCTs (24, 25). A dynamic MRR containing various copy numbers of IS*26*-*bla*_CTX-M-65_ and IS*26*-*fosA3* and corresponding circle intermediates has been reported in the E. coli plasmid IncZ-7 (25). In our study, MRRs containing multiple IS*26*-flanked PCTs and various IS*26*-derived circular intermediates were detected in *bla*_NDM_-carrying IncB/O/K/Z plasmids. Harmer and Hall recently described the mobility of IS*26* in detail and demonstrated that replicative transposition driven by this element generates circular molecules containing one copy of IS*26* and an adjacent DNA segment carrying an antibiotic resistance (or other) gene, which they designated a “translocatable unit” (TU) (26). However, some reports suggest that a TU cannot be formed by replicative transposition when tandem arrays of a resistance gene are present with an IS*26* between them (27) or when structures share an IS*26* between two compound transposons (28). Furthermore, although in the absence of an active homologous recombination system, excision of TU was demonstrated in the context of Tn*4352B* when it was adjacent to the two G residues at the left end of the IS*26*; in other cases, this does not occur (29), and generation of a TU from a preexisting transposon would necessarily occur via homologous recombination (26). RecA-dependent simple homologous recombination from a IS*26*-based PCT could lead to excision for forming the TU (30, 31). In our study, the absence of GG at the left end of the IS*26* and target site duplication (TDS) flanking IS*26* suggest that the circular intermediates were most likely generated from preexisting tandem arrays of IS*26*-associated modules on MRR via homologous recombination in the *recA*-carrying (*recA^+^*) E. coli strain J53. Importantly, the different TU intermediates observed in a single strain in our study may reflect a dynamic process involving the insertion and deletion of a transposable unit by homologous recombination.

Fusion between plasmids during recombination or cointegration occurs frequently, further extending the resistance profiles of pathogens and broadening the host spectrum of the fusion plasmid (7). In this study, sequence analysis of pNDM33-1, pNDM33-2, and pNDM-TJ33 enabled us to shed light on IS*26* mobility via homologous recombination. The sites on pNDM-TJ33 (A1 and A2), which was formed by fusion of the IncHI2 and IncI1 plasmids, suggest that the presence of homologous sequences IS*26*, streptomycin 3′-adenylyltransferase, and a truncated *IntI1* gene in both the IncI1 and IncHI2 plasmids could facilitate plasmid fusion (Fig. 3b). Based on analysis of site B on pNDM-TD33, we speculate that the MRR from pNDM-TJ33 transferred to pNDM33-2 during an intramolecular homologous recombination event, forming a new plasmid (pNDM-TD33) (Fig. 3b). It seems likely that the presence of homologous IS*26* sequences may facilitate the formation of various plasmid types and accelerate the evolution of antibiotic resistance plasmids.

Intermolecular transposition can help plasmids acquire new genes, while intramolecular recombination accompanied by deletion can streamline resistance gene clusters by removing redundant or metabolically costly genes (24) and/or by creating new hybrid promoters to adjust the expression of remaining genes (32, 33). It has been reported that, if IS*26* transposition remodels plasmid-borne genes involved in conjugational transfer and plasmid stability, the plasmid may then need to acquire new mechanisms to rectify these effects, resulting in new biological characteristics (24). In our study, we found that pNDM-TD33 had greater stability and a significant increase in fitness compared with pNDM33-1 and pNDM-TJ33, indicating that it is more capable of being transferred by conjugation and persisting within the host bacterium. The plasmid evolution process described in our study provides an example of how novel *bla*_NDM_-harboring plasmids with characteristics that help them spread widely can emerge.

In conclusion, we describe the generation of different MRRs by IS*26*-flanked PCTs that also give rise to various circular intermediates, thus providing important insight into contribution of IS*26* to MRRs on plasmids which carry *bla*_NDM_, and consequently into the rapid dissemination of NDM among *Enterobacteriaceae*. Furthermore, our study provides direct evidence of plasmid evolution and highlights the importance of homologous recombination in the evolution and diversity of carbapenem resistance plasmids when IS*26* is present. While fusion between conjugative plasmids has been reported previously, our work is the first to demonstrate the dynamic process by which IS*26* facilitates fusion of *bla*_NDM-5_-harboring plasmids. These data could be used proactively to assist the poultry industry in China in developing food safety measures designed to limit the transmission of these NDM-carrying isolates by food animals.

## MATERIALS AND METHODS

### Strains and antimicrobial susceptibility testing.

From August 2016 to December 2017, a total of 470 nonduplicate samples were collected from five chicken and one duck farms in two provinces of China (Fig. 1; see also Table S1 in the supplemental material). Briefly, fecal samples were collected from randomly selected ducks (*n* = 77) and chickens (*n* = 393). Swabs of feces were inoculated into sterile selenite cystine broth and incubated for 24 h at 37°C, after which the culture was streaked to chromogenic medium selective for E. coli (CHROMagar Microbiology, France) and incubated for another 24 h at 37°C. One blue colony was selected from each plate and confirmed to be E. coli by matrix-assisted laser desorption ionization–time of flight mass spectrometry (MALDI-TOF MS). The carbapenem-resistant isolates were screened for the *bla*_NDM_ gene by PCR using previously reported primers (34) and by sequencing (35).

The MICs of antibiotics were determined using the agar dilution method (36), and the results were interpreted following Clinical and Laboratory Standards Institution (CLSI) guidelines (M100-S25) (37) and veterinary CLSI guidelines (VET01-A4/VET01-S2) (38). The following twenty antimicrobial agents were tested: amoxicillin, ceftiofur, ceftazidime, cefotaxime, imipenem, ertapenem, meropenem, aztreonam, gentamicin, amikacin, tetracycline, doxycycline, tigecycline, chloramphenicol, florfenicol, ciprofloxacin, polymyxin E, fosfomycin, trimethoprim-sulfamethoxazole, and trimethoprim. E. coli ATCC 25922 served as the quality control strain.

### Molecular typing.

All *bla*_NDM_-positive E. coli isolates were classified according to XbaI pulsed-field gel electrophoresis (PFGE) typing, as previously described (39). Comparison of PFGE patterns was performed with BioNumerics v.7.1 software (Applied Maths, Sint-Martens-Latem, Belgium) using the Dice 85% similarity coefficient.

### Transferability of *bla*_NDM_.

Conjugation experiments were performed using sodium azide-resistant E. coli J53 as the recipient (39). Transconjugants were selected on MacConkey agar containing 150 mg/liter sodium azide and 0.5 mg/liter meropenem. For isolates where no transconjugants were obtained, an alternative E. coli (DH5α) was used as a recipient for transformation experiments (40), and transformants were selected for on Luria-Bertani (LB) agar containing 0.5 mg/liter meropenem. The presence of *bla*_NDM_ in transconjugants/transformants was further confirmed by PCR and sequencing.

### Plasmids and genetic context of *bla*_NDM_.

The replicon types for all transconjugants and transformants with *bla*_NDM_-carrying plasmids were determined by PCR-based replicon typing (PBRT) as previously described (41). All transconjugants and transformants were subjected to S1-PFGE and Southern blotting (42) using digoxigenin-labeled probes specific for the *bla*_NDM_ gene.

Plasmid DNA was purified using a Qiagen plasmid midi kit (Qiagen, Germany). The predominant plasmid was completely sequenced using Illumina HiSeq technology. Sequence reads were assembled into contigs using SOAPdenovo v.2.04. Whole-genome sequencing of strain GD33 was performed using an Illumina platform and a PacBio Sequel single-molecule real-time (SMRT) sequencing platform (Novogene, Beijing). *De novo* PacBio Sequel read assemblies were generated using SMRT Link v.5.0.1, and the sequences were optimized by Illumina reads using Burrows-Wheeler Aligner (BWA) v.0.7.8. Whole-genome sequencing of strains TJ33 and TD33 was performed using an Oxford Nanopore GridION platform and an Illumina platform (NextOmics, Wuhan, China). The Nanopore reads were assembled using Canu 1.7.11, and the sequences were optimized by Illumina reads using BWA V0.7.17 and Pilon V1.22.

The plasmid sequences were annotated using RAST (http://rast.nmpdr.org/rast.cgi) and BLAST (http://blast.ncbi.nlm.nih.gov/Blast.cgi) (43). ARGs and plasmid replicon types were identified using ResFinder (https://cge.cbs.dtu.dk/services/ResFinder/) and PlasmidFinder (https://cge.cbs.dtu.dk/services/PlasmidFinder/), respectively. Mobile elements were identified using ISfinder (https://www-is.biotoul.fr/). Specific primers used for further analysis of these plasmids were designed using Primer Premier v.5.0 (see Table S2 in the supplemental material). Target sequences were identified using gradient temperature PCR, and amplicon identities were confirmed by Sanger sequencing.

### Plasmid stability and fitness.

Two transconjugants (TJ33 and TD33) were chosen for competition and plasmid stability experiments, as previously described (44, 45). These strains were incubated for 8 days for stability assays and for 12 days for competition assays. Luria-Bertani agar plates containing meropenem (1 μg/ml) were used for the stability experiment, and Luria-Bertani agar plates containing cefotaxime (1 μg/ml) were used for the competition experiment. Plasmid stability was assessed by the percentage of strains that retained plasmids, and the fitness of the strains was assessed by measuring the relative fitness cost (W), as follows: a W of >1 means the plasmid-harboring strains were more fit, while a W of <1 means the host strains used as a negative control were more fit.

### Data availability.

The sequencing data of the plasmids have been deposited in GenBank under the following accession numbers: MN335919 (pNDM-T2), MN335921 (pNDM-T6), MN335922 (pNDM-T16), MN915011 (pNDM33-1), MN915012 (pNDM33-2), CP076650 (pNDM33-3), CP076649 (pNDM33-4), CP076646 (GD33 chromosome), MN915010 (pNDM-TJ33), and MN915013 (pNDM-TD33).
