# Supplementary material for: IS26 Is Responsible for the Evolution and Transmission of blaNDM-Harboring Plasmids in Escherichia coli of Poultry Origin in China
Source: mSystems. 2021 Jul 13;6(4):e00646-21. doi: 10.1128/mSystems.00646-21 (PMC8407110; doi:10.1128/mSystems.00646-21)
Supplement: TABLE S1 [file msystems.00646-21-st001.docx]

**Supplementary material**

**Table S1** MIC of *bla*_NDM_-harboring *E. coli* to different antibiotics

| Isolates | Year | Province | Farm^a^ | Resistance genes | Resistance phenotype^b^ | | | | | | | | | | | | | | | | | | | | | |
| --- | --- | --- | --- | --- | --- | --- | --- | --- | --- | --- | --- | --- | --- | --- | --- | --- | --- | --- | --- | --- | --- | --- | --- | --- | --- | --- |
|  |  |  |  |  | AMX | CTF | CAZ | CTX | CTX/EDTA | IPM | ETP | MEM | MEM/EDTA | ATM | GEN | AMK | TET | DOX | TGC | CHL | FFC | CIP | CL | FOF | SXT | TMP |
| GD1 | 2016 | SD | SC-1 | *bla*_NDM-5_, *bla*_CTX-M_, *bla*_TEM_, *fosA3* | 512 | >512 | >512 | >512 | 512 | 16 | 8 | 32 | 0.25 | 16 | 8 | 1 | 64 | 16 | 0.5 | 256 | 512 | 32 | 0.25 | >1024 | 8 | 32 |
| GD2 | 2016 | SD | SC-1 | *bla*_NDM-9_, *bla*_CTX-M_, *mcr-1*, *fosA3* | 512 | >512 | >512 | >512 | 256 | 4 | 8 | 2 | 0.06 | 32 | 128 | 1 | 128 | 16 | 0.5 | 256 | 512 | 4 | 8 | >1024 | 8 | 32 |
| GD3 | 2016 | SD | SC-1 | *bla*_NDM-9_, *bla*_CTX-M_, *bla*_TEM_, *mcr-1* | 512 | >512 | >512 | >512 | 256 | 16 | 4 | 16 | 0.125 | 16 | 64 | 2 | 64 | 16 | 0.25 | 256 | 128 | 32 | 16 | 0.5 | 8 | 32 |
| GD4 | 2016 | SD | SC-1 | *bla*_NDM-5_, *bla*_CTX-M_, *bla*_TEM_, *fosA3* | 512 | >512 | >512 | >512 | 512 | 32 | 16 | 32 | 0.25 | 16 | 8 | 1 | 64 | 16 | 1 | 128 | 512 | 32 | 0.25 | >1024 | 8 | 32 |
| GD5 | 2016 | SD | SC-1 | *bla*_NDM-1_, *bla*_CTX-M_, *bla*_TEM_, *mcr-1*, *fosA3*, *rmtB* | 512 | >512 | >512 | >512 | 512 | 16 | 16 | 8 | 0.06 | 256 | 512 | >512 | 64 | 16 | 0.5 | 256 | 512 | 32 | 8 | >1024 | 8 | 32 |
| GD6 | 2016 | SD | SC-1 | *bla*_NDM-9_, *bla*_CTX-M_, *bla*_TEM_, *mcr-1* | 512 | >512 | >512 | >512 | 256 | 16 | 4 | 4 | 0.06 | 16 | 64 | 1 | 128 | 16 | 0.25 | 64 | 256 | 16 | 16 | 1 | 8 | 32 |
| GD7 | 2016 | SD | SC-1 | *bla*_NDM-5_, *bla*_CTX-M_, *bla*_TEM_, *fosA3* | 512 | >512 | >512 | >512 | 512 | 16 | 8 | 32 | 0.25 | 16 | 8 | 1 | 64 | 16 | 1 | 256 | 512 | 32 | 0.25 | >1024 | 8 | 32 |
| GD8 | 2016 | SD | SC-1 | *bla*_NDM-9_, *bla*_CTX-M_, *bla*_TEM,_ *mcr-1* | 512 | >512 | >512 | >512 | 128 | 16 | 4 | 8 | 0.06 | 16 | 64 | 2 | 64 | 16 | 0.5 | 128 | 128 | 16 | 16 | 2 | 8 | 32 |
| GD9 | 2016 | SD | SC-1 | *bla*_NDM-5_, bla_CTX-M_, *bla*_TEM_, *fosA3* | 512 | >512 | >512 | >512 | 512 | 16 | 8 | 32 | 0.25 | 16 | 8 | 1 | 64 | 16 | 1 | 256 | 512 | 32 | 0.25 | >1024 | 8 | 32 |
| GD11 | 2017 | GD | GC-1 | *bla*_NDM-5_, *bla*_CTX-M_, *qnrS*, *oqxA* | 512 | 256 | >512 | 512 | 256 | 32 | 32 | 8 | 0.125 | 32 | 0.5 | 1 | 256 | 16 | 2 | >512 | >512 | 4 | 2 | 0.5 | 8 | 32 |
| GD12 | 2017 | GD | GC-1 | *bla*_NDM-5_, *bla*_CTX-M_, *qnrS* | 512 | 256 | >512 | 256 | 64 | 8 | 4 | 2 | 0.125 | 4 | 4 | 1 | 128 | 16 | 0.5 | 128 | 256 | 8 | 0.25 | 1 | 8 | 32 |
| GD13 | 2017 | GD | GC-1 | *bla*_NDM-5_, *bla*_CTX-M_, *qnrS* | 512 | 256 | >512 | 256 | 64 | 8 | 4 | 4 | 0.125 | 4 | 4 | 1 | 128 | 16 | 0.5 | 128 | 128 | 8 | 0.25 | 1 | 8 | 32 |
| GD14 | 2017 | GD | GC-1 | *bla*_NDM-5_, *bla*_CTX-M_, *bla*_TEM_, *qnrS* | 512 | 256 | >512 | 256 | 64 | 8 | 4 | 4 | 0.06 | 4 | 4 | 1 | 128 | 16 | 0.5 | 128 | 256 | 8 | 0.25 | 1 | 8 | 32 |
| GD15 | 2017 | GD | GD-1 | *bla*_NDM-5_, *bla*_TEM_, *oqxA*, *mcr-1* | 512 | 256 | >512 | 256 | 256 | 16 | 8 | 8 | 0.25 | 0.125 | 32 | 1 | 256 | 32 | 1 | 512 | >512 | 32 | 8 | 1 | 8 | 32 |
| GD16 | 2016 | SD | SC-1 | *bla*_NDM-5_, *bla*_CTX-M_, *qnrB*, *aac-(6′)-Ib-cr*, *oqxA*, *fosA3* | 512 | 512 | >512 | >512 | 256 | 16 | 32 | 8 | 0.25 | 64 | 1 | 2 | 128 | 32 | 0.5 | 512 | >512 | 2 | 0.25 | 1024 | 8 | 32 |
| GD17 | 2017 | GD | GD-1 | *bla*_NDM-5_, *bla*_CTX-M_, *bla*_TEM_, *oqxA*, *fosA3* | 512 | 256 | >512 | 512 | 128 | 16 | 16 | 16 | 0.25 | 32 | 0.5 | 2 | 128 | 32 | 1 | 256 | >512 | 32 | 0.25 | >1024 | 8 | 32 |
| GD18 | 2017 | GD | GD-1 | *bla*_NDM-5_, *bla*_TEM_, *qnrS*, *oqxA* | 512 | 512 | >512 | 512 | 256 | 32 | 32 | 8 | 0.125 | 32 | 64 | 1 | 256 | 64 | 4 | >512 | 256 | 4 | 0.25 | 0.5 | 8 | 32 |
| GD19 | 2017 | GD | GD-1 | *bla*_NDM-5_, *bla*_TEM_, *oqxA*, *mcr-1* | 512 | 256 | >512 | 256 | 64 | 16 | 8 | 8 | 0.25 | 0.06 | 64 | 1 | 256 | 32 | 1 | >512 | >512 | 32 | 8 | 1 | 8 | 32 |
| GD20 | 2017 | GD | GD-1 | *bla*_NDM-5_, *qnrS* | 512 | 256 | >512 | 512 | 32 | 8 | 8 | 4 | 0.06 | 0.125 | 0.5 | 2 | 256 | 64 | 2 | 256 | >512 | 16 | 0.25 | 0.5 | 8 | 32 |
| GD21 | 2016 | SD | SC-1 | *bla*_NDM-5_, *bla*_TEM_, *oqxA*, *mcr-1* | 512 | 256 | >512 | 256 | 16 | 16 | 8 | 8 | 0.25 | 0.125 | 64 | 1 | 256 | 32 | 1 | 512 | >512 | 32 | 8 | 1 | 8 | 32 |
| GD22 | 2016 | SD | SC-1 | *bla*_NDM-5_, *bla*_TEM_, *oqxA*, *mcr-1* | 512 | 256 | >512 | 256 | 16 | 16 | 8 | 8 | 0.25 | 0.06 | 32 | 1 | 256 | 32 | 1 | >512 | >512 | 32 | 8 | 1 | 8 | 32 |
| GD27 | 2017 | GD | GD-1 | *bla*_NDM-5_, *bla*_TEM_, *qnrS* | 512 | 256 | >512 | 256 | 32 | 8 | 2 | 4 | 0.125 | 4 | 8 | 1 | 256 | 128 | 0.25 | 128 | 128 | 0.125 | 0.25 | 0.5 | 8 | 32 |
| GD28 | 2017 | GD | GD-1 | *bla*_NDM-5_, *bla*_TEM_, *qnrD*, *qnrS*, *aac-(6′)-Ib-cr*, *mcr-1* | 512 | 256 | >512 | 512 | 32 | 8 | 8 | 8 | 0.125 | 4 | 0.5 | 1 | 256 | 64 | 2 | 256 | >512 | 2 | 8 | 1 | 8 | 32 |
| GD30 | 2017 | GD | GD-1 | *bla*_NDM-5_, *bla*_CTX-M_, *bla*_TEM_, *qnrS*, *oqxA*, *fosA3* | 512 | 256 | >512 | 256 | 32 | 8 | 16 | 16 | 0.125 | 0.5 | 4 | 1 | 128 | 32 | 0.5 | 512 | 512 | 16 | 0.25 | 256 | 8 | 32 |
| GD32 | 2016 | SD | SC-1 | *bla*_NDM-5_, *bla*_CTX-M_, *bla*_TEM_, *qnrS* | 512 | 512 | >512 | 512 | 128 | 8 | 16 | 8 | 0.125 | 32 | 32 | 2 | 256 | 64 | 2 | 128 | 512 | 32 | 0.25 | 1 | 8 | 32 |
| GD33 | 2017 | GD | GD-1 | *bla*_NDM-5_, *bla*_CTX-M_, *bla*_TEM_, *qnrS*, *oqxA*, *fosA3*, *rmtB* | 512 | 256 | >512 | 512 | 64 | 16 | 64 | 16 | 0.5 | 8 | >512 | >512 | 256 | 64 | 2 | >512 | >512 | 64 | 0.25 | >1024 | 8 | 32 |
| GD36 | 2017 | GD | GC-2 | *bla*_NDM-5_, *bla*_CT_X-M, *bla*_TEM_, *fosA3*, *qnrS* | 512 | 512 | >512 | 512 | 128 | 16 | 8 | 8 | 0.125 | 16 | 8 | 1 | 64 | 16 | 0.5 | 256 | 128 | 16 | 0.25 | >1024 | 8 | 32 |
| GD37 | 2017 | GD | GC-3 | *bla*_NDM-5_, *bla*_CTX-M_ | 256 | >512 | >512 | >512 | >512 | 8 | 32 | 16 | 0.125 | 128 | 0.25 | 1 | 64 | 8 | 0.25 | 64 | 128 | 8 | 0.25 | 1 | 8 | 32 |
| SDA | 2017 | GD | GC-1 | *bla*_NDM-9,_ *aac-(6′)-Ib-cr, oqxA, fosA3* | 256 | 256 | >512 | 256 | 16 | 16 | 8 | 4 | 0.125 | 0.125 | 8 | 2 | 128 | 64 | 1 | 512 | >512 | 64 | 0.25 | >1024 | 8 | 32 |
| SDB | 2017 | GD | GC-1 | *bla*_NDM-9_, bla_OXA_, aac-(6′)-Ib-cr, oqxA, fosA3 | 256 | 256 | >512 | 256 | 8 | 8 | 8 | 8 | 0.125 | 0.125 | 8 | 4 | 256 | 64 | 1 | 512 | >512 | 64 | 0.25 | >1024 | 8 | 32 |
| SDC | 2017 | GD | GC-1 | *bla*_NDM-9_, *bla*_OXA_, *aac-(6′)-Ib-cr*, *oqxA*, *fosA3* | 512 | 256 | >512 | 256 | 16 | 8 | 8 | 8 | 0.06 | 0.125 | 8 | 4 | 128 | 64 | 1 | 512 | >512 | 64 | 0.25 | >1024 | 8 | 32 |
| SDD | 2017 | GD | GC-1 | *bla*_NDM-9_, *bla*_OXA_, *aac-(6′)-Ib-cr*, *oqxA*, *fosA3* | 512 | 256 | >512 | 256 | 16 | 16 | 8 | 8 | 0.06 | 0.125 | 8 | 4 | 128 | 64 | 1 | 512 | 512 | 64 | 0.25 | >1024 | 8 | 32 |
| SDE | 2017 | GD | GC-1 | *bla*_NDM-5_, *bla*_CTX-M_, *bla_T_*_EM_, *qnrB*, *qnrS* | 512 | 256 | >512 | 256 | 64 | 8 | 4 | 4 | 0.125 | 4 | 8 | 1 | 128 | 32 | 0.5 | 128 | 128 | 32 | 0.25 | 4 | 8 | 32 |
| TJ33 | - | - | - | *bla*_NDM-5_、 *floR*、 *qnrS* | 512 | 256 | >512 | 256 | 16 | 8 | 16 | 8 | 0.125 | 0.06 | 4 | 0.5 | 128 | 16 | 0.5 | 256 | 256 | 4 | 0.125 | 1 | >16 | 32 |
| TD33 | - | - | - | *bla*_NDM-5_、 *qnrS* | 512 | 128 | 512 | 128 | 4 | 4 | 4 | 2 | 0.06 | 0.03 | 8 | 0.25 | 1 | 1 | 0.125 | 8 | 8 | 2 | 0.125 | 0.5 | 0.125 | 1 |
| pNDM33-1 | - | - | - | *bla*_NDM-5_  *qnrS* | 512 | 256 | >512 | 256 | 8 | 4 | 16 | 8 | 0.125 | 0.06 | 4 | 0.25 | 128 | 16 | 0.5 | 8 | 8 | 2 | 0.125 | 1 | 16 | 32 |
| pNDM33-2 | - | - | - | *floR* | 512 | 0.5 | 0.125 | 0.06 | 0.03 | 0.015 | 0.015 | 0.06 | 0.06 | 0.03 | 2 | 0.25 | 1 | 1 | 0.125 | 256 | 256 | 0.125 | 0.125 | 0.5 | 0.125 | 2 |
| pNDM-T2 | - | - | - | *bla*_NDM-9_、 *fosA3* | 512 | 128 | 512 | 64 | 2 | 2 | 4 | 2 | 0.06 | 0.06 | <0.125 | 0.25 | 1 | 1 | 0.125 | 8 | 2 | 0.008 | 0.125 | 256 | 16 | 32 |
| pNDM-T6 | - | - | - | *bla*_NDM-9_ | 5122 | 128 | 512 | 64 | 2 | 4 | 4 | 2 | 0.06 | 0.06 | <0.125 | 0.25 | 1 | 1 | 0.125 | 8 | 2 | 0.008 | 0.125 | 1 | >16 | 32 |
| pNDM-T16 | - | - | - | *bla*_NDM-5_ | 5122 | 128 | 512 | 128 | 4 | 4 | 4 | 2 | 0.06 | 0.06 | <0.125 | 0.25 | 1 | 1 | 0.125 | 8 | 2 | 0.008 | 0.125 | 1 | 0.03 | 2 |
| J53 | - | - | - | - | 2 | 0.5 | 0.125 | 0.06 | 0.06 | 0.06 | 0.015 | 0.03 | 0.03 | 0.06 | 0.25 | 0.5 | 0.5 | 2 | 0.5 | 2 | 8 | 0.008 | 0.125 | 1 | 0.06 | 0.5 |
| C600 | - | - | - | - | 2 | 0.25 | 0.125 | 0.06 | 0.03 | 0.06 | 0.015 | 0.06 | 0.03 | 0.06 | 1 | 2 | 1 | 0.125 | 0.5 | 4 | 0.03 | 2 | 0.125 | 0.06 | 1 | 2 |
| DH5α | - | - | - | - | 4 | 0.25 | 0.06 | 0.03 | 0.015 | 0.06 | 0.015 | 0.015 | 0.015 | 0.06 | 0.0125 | 0.25 | 0.5 | 1 | 0.125 | 2 | 2 | 0.015 | 0.125 | 0.25 | 0.06 | 1 |
| ATCC  25922 | - | - | - | - | 4 | 0.25 | 0.125 | 0.06 | 0.03 | 0.125 | 0.008 | 0.03 | 0.015 | 0.5 | 0.5 | 1 | 1 | 0.5 | 0.125 | 4 | 2 | 0.008 | 0.5 | 1 | 0.25 | 1 |

^a^ SC: farm of chicken in Shandong province; GC: farm of chicken in Guangdong province; GD: farm of duck in Guangdong province.

^b^ AMX: Amoxicillin. CTF: Ceftiofur. CAZ: Ceftazidime. CTX: Cefotaxime. CTX+EDTA: Cefotaxime + EDTA70 μg/mL. IPM: Imipenem. ETP: Ertapenem. MEM: Meropenem. MEM+EDTA: Meropenem + EDTA 70 μg/mL. ATM: Aztreonam. GEN: Gentamycin. AMK: Amikacin. TET: Tetracycline. DOX: Doxycycline. TGC: Tigecycline. CHL: Chloramphenicol. FFC: Florfenicol. CIP: Ciprofloxacin. CL: Polymyxin E. FOF: Fosfomycin. SXT: Trimethoprim-sulfamethoxazole (1:19). TMP: Trimethoprim
