# Supplementary figures and images for: IS26 Is Responsible for the Evolution and Transmission of blaNDM-Harboring Plasmids in Escherichia coli of Poultry Origin in China
Source: mSystems. 2021 Jul 13;6(4):e00646-21. doi: 10.1128/mSystems.00646-21 (PMC8407110; doi:10.1128/mSystems.00646-21)

**Supplementary material**


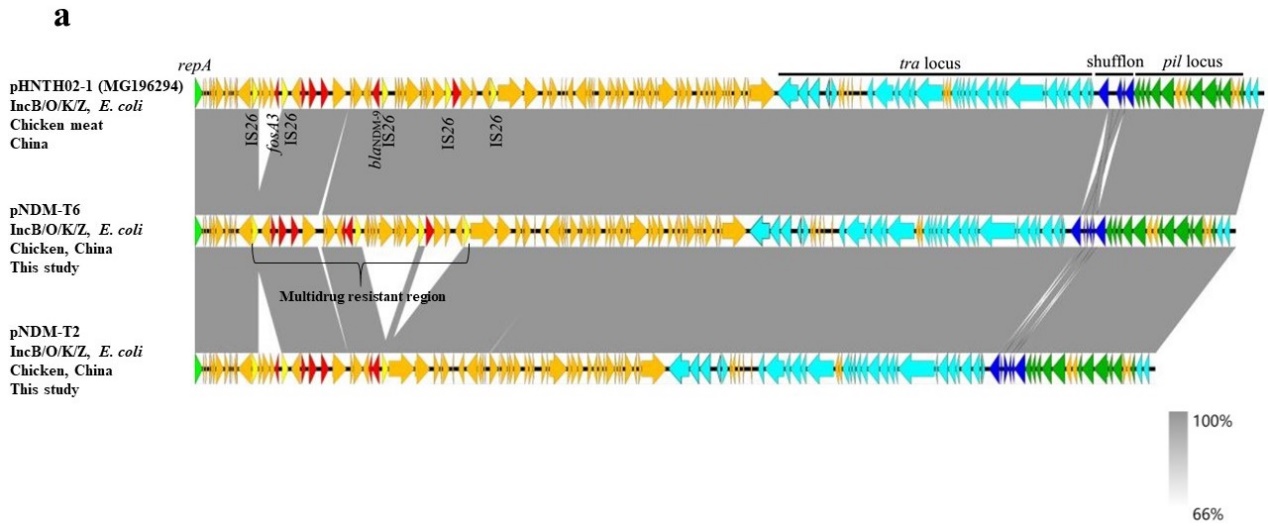


**Figure S1** Sequence alignment of *bla*NDM-9-positive IncB/O/K/Z plasmids.

Supplement: FIG S1 [file msystems.00646-21-sf001.docx]

**Supplementary material**


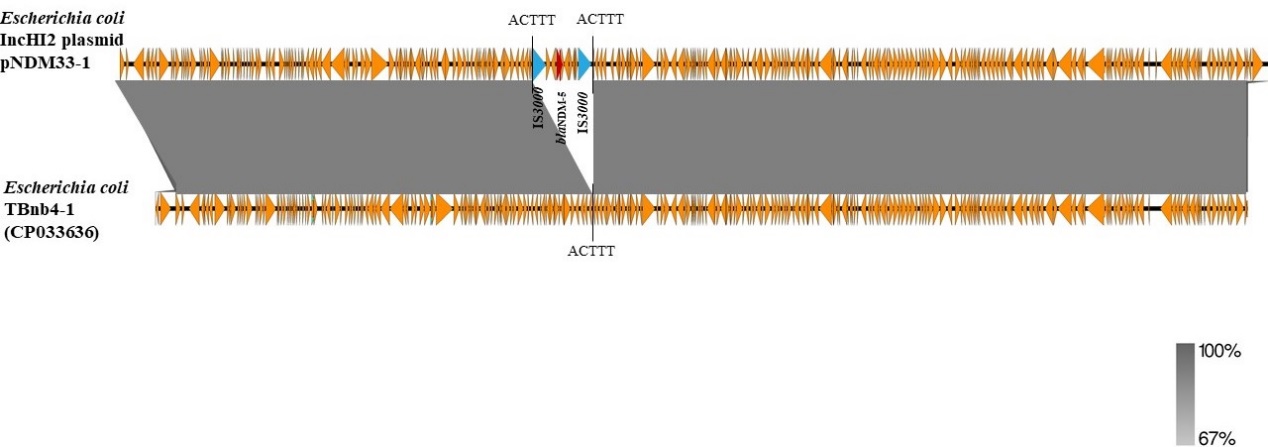


**Figure S2** Sequence alignment of *bla*NDM-5-positive IncHI2 plasmids.

Supplement: FIG S2 [file msystems.00646-21-sf002.docx]
