# Supplementary material for: IS26 Is Responsible for the Evolution and Transmission of blaNDM-Harboring Plasmids in Escherichia coli of Poultry Origin in China
Source: mSystems. 2021 Jul 13;6(4):e00646-21. doi: 10.1128/mSystems.00646-21 (PMC8407110; doi:10.1128/mSystems.00646-21)
Supplement: TABLE S2 [file msystems.00646-21-st002.docx]

**Supplementary material**

**Table S2**. Primers used to detect *bla*_NDM_-harboring transconjugants

| Primer | Nucleotide sequence (5′–3′) | Product length (bp) | Reference |
| --- | --- | --- | --- |
| Circle A | P1: TGGCGGCGAAAGTCAG | 2394 | This study |
|  | P2: AGGCGAGCATCGTTTGTT |  |  |
| Circle B | P3: AGATGATTCACGACGAACTGC | 1513 | This study |
|  | P4: CGCAGCGGTTACGACATT |  |  |
| Circle C | P5: GGTGACCATCCCGATACTTG | 2473 | This study |
|  | P6: CGCAGCGGTTACGACATT |  |  |
| Circle D | P7: TGGCGGCGAAAGTCAG | 2394 | This study |
|  | P8: AGGCGAGCATCGTTTGTT |  |  |
| Circle E | P9: AGATTGCCGAGCGACTTG | 2441 | This study |
|  | P10: GAGACGGTGACGATGATGTTA |  |  |
| Circle F | P11: GCTGCTCGTTCGGCTAT | 1783 | This study |
|  | P12: CGACTGGACCTTCCTTCTG |  |  |
| *bla*_NDM_ | F: GGTTTGGCGATCTGGTTTTC | 621 | (1) |
|  | R: CGGAATGGCTCATCACGATC |  |  |
| ERIC | F: ATGTAAGCTCCTGGGGATTCAC | N.A ^a^ | (2) |
|  | R: AAGTAAGTGACTGGGGTGAGCG |  |  |
| IncHI2 | F: TTTCTCCTGAGTCACCTGTTAACAC | 644 | (3) |
|  | R: GGCTCACTACCGTTGTCATCCT |  |  |
| IncI1 | F: CGAAAGCCGGACGGCAGAA | 139 | (3) |
|  | R: TCGTCGTTCCGCCAAGTTCGT |  |  |
| Site A1 | F: AGGTTGTGGGATTGTCTTG | 3662 | This study |
|  | R: TGGTGAGTAGAGTTTCAGGGT |  |  |
| Site A2 | F: AGTCTTCCCTTGTTATTGTG | 1689 | This study |
|  | R: TCCGTGAGCGGTGAT |  |  |
| Site B | F: AGGCGATTTGATCTGCA | 2011 | This study |
|  | R: AATGAACCGAGCACCAG |  |  |

^a^ N.A, not applicable

**REFERENCES**

1. Poirel L, Walsh TR, Cuvillier V, Nordmann P. 2011. Multiplex PCR for detection of acquired carbapenemase genes. Diagn Microbiol Infect Dis 70:119-123.

2. Versalovic J, Koeuth T, Lupski JR. 1991. Distribution of repetitive DNA sequences in eubacteria and application to fingerprinting of bacterial genomes. Nucleic Acids Res 19:6823-6831.

3. Carattoli A, Bertini A, Villa L, Falbo V, Hopkins KL, Threlfall EJ. 2005. Identification of plasmids by PCR-based replicon typing. J Microbiol Methods 63:219-228.
