# Supplementary material for: IS26 Is Responsible for the Evolution and Transmission of blaNDM-Harboring Plasmids in Escherichia coli of Poultry Origin in China
Source: mSystems. 2021 Jul 13;6(4):e00646-21. doi: 10.1128/mSystems.00646-21 (PMC8407110; doi:10.1128/mSystems.00646-21)
Supplement: FIG S3 [file msystems.00646-21-sf003.docx]

**Supplementary material**


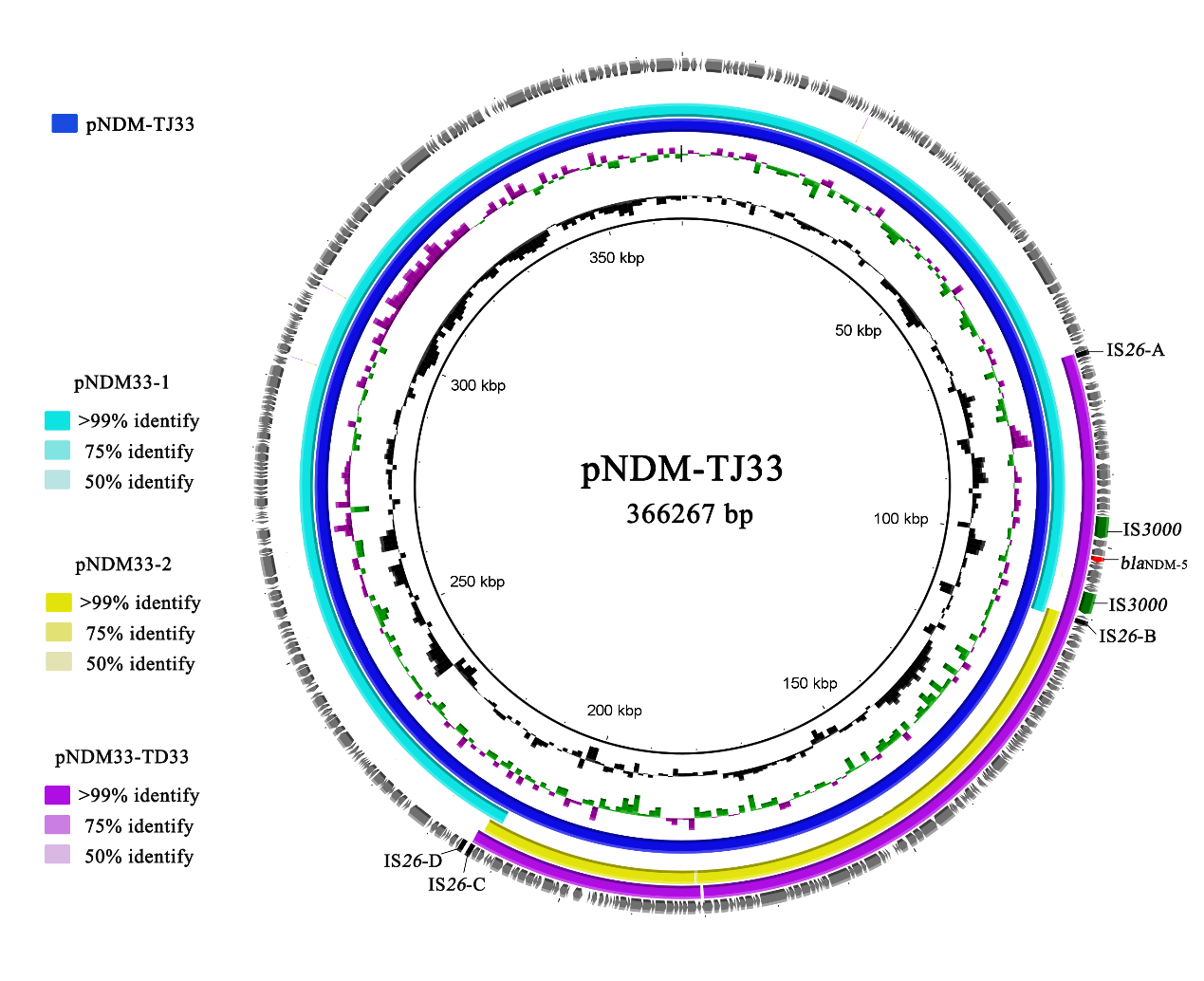


**Figure S3** Pairwise BLASTn alignment of pNDM33-1, pNDM33-2, pNDM-TJ33 and pNDM-TD33 performed using BRIG (1).

**REFERENCES**

1. Alikhan, N.F., Petty, N.K., Ben, Zakour. N.L., Beatson, S.A., 2011. BLAST Ring Image Generator (BRIG): simple prokaryote genome comparisons. BMC. Genomics. 12: 402.
