# Supplementary material for: IS26 Is Responsible for the Evolution and Transmission of blaNDM-Harboring Plasmids in Escherichia coli of Poultry Origin in China
Source: mSystems. 2021 Jul 13;6(4):e00646-21. doi: 10.1128/mSystems.00646-21 (PMC8407110; doi:10.1128/mSystems.00646-21)
Supplement: TABLE S3 [file msystems.00646-21-st003.docx]

**Supplementary material**

**Table S3** Distribution of resistance genes and replicons in four plasmids related to plasmid inter and intramolecular recombination

| Plasmid name | | | pNDM33-1 | pNDM33-2 | pNDM-TJ33 | pNDM-TD33 |
| --- | --- | --- | --- | --- | --- | --- |
| Host strains | | | GD33 | GD33 | TJ33 | TD33 |
| Size (bp) | | | 266, 777 | 113, 068 | 366, 267 | 141, 890 |
| Replicons | | IncHI2 |  |  |  |  |
|  |  | IncHI2A |  |  |  |  |
|  |  | IncI1 |  |  |  |  |
| Resistance  genes | Aminoglycosides | *aadA1* |  |  |  |  |
|  |  | *aadA2b* |  |  |  |  |
|  |  | *aadA22* |  |  |  |  |
|  |  | *aph(3')-Ia* |  |  |  |  |
|  |  | *aph(3'')-Ib* |  |  |  |  |
|  |  | *aac(3)-IV* |  |  |  |  |
|  |  | *aph(4)-Ia* |  |  |  |  |
|  |  | *aph(6)-Id* |  |  |  |  |
|  | Beta-lactams | *bla*_NDM-5_ |  |  |  |  |
|  |  | *bla*_OXA-10_ |  |  |  |  |
|  |  | *bla*_TEM-1B_ |  |  |  |  |
|  | Quinolones | *qnrS1* |  |  |  |  |
|  | Phenicols | *floR* |  |  |  |  |
|  |  | *cmlA1* |  |  |  |  |
|  | Rifampicin | *ARR-2* |  |  |  |  |
|  | Sulphonamides | *sul3* |  |  |  |  |
|  | Tetracyclines | *tet(A)* |  |  |  |  |
|  | Trimethoprim | *dfrA14* |  |  |  |  |
|  | Macrolides | *lnu(F)* |  |  |  |  |
|  |  | *erm(B)* |  |  |  |  |
|  |  | *mph(A)* |  |  |  |  |

^a^ Rectangles marked in red indicate the presence of the corresponding genes listed on the left.
