# Supplementary material for: IS26 Is Responsible for the Evolution and Transmission of blaNDM-Harboring Plasmids in Escherichia coli of Poultry Origin in China
Source: mSystems. 2021 Jul 13;6(4):e00646-21. doi: 10.1128/mSystems.00646-21 (PMC8407110; doi:10.1128/mSystems.00646-21)
Supplement: FIG S4 [file msystems.00646-21-sf004.docx]

**Supplementary material**


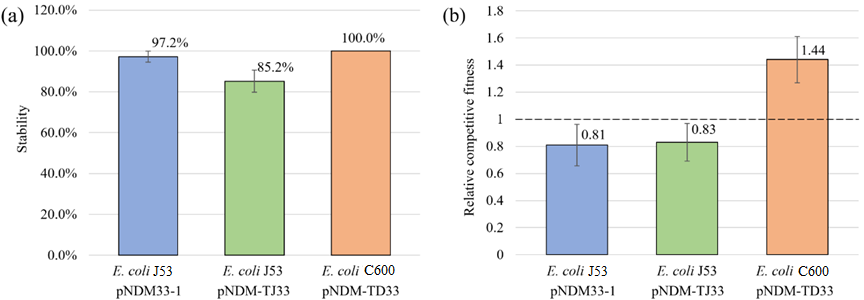


**Figure S4** Effects of pNDM33-1, pNDM-TJ33 and pNDM-TD33 on *E. coli* host strains. (a) Plasmid stability. (b) Competition.
